# Lipid and DHA-production in *Aurantiochytrium* sp. – Responses to nitrogen starvation and oxygen limitation revealed by analyses of production kinetics and global transcriptomes

**DOI:** 10.1038/s41598-019-55902-4

**Published:** 2019-12-19

**Authors:** Tonje M. B. Heggeset, Helga Ertesvåg, Bin Liu, Trond E. Ellingsen, Olav Vadstein, Inga Marie Aasen

**Affiliations:** 1 SINTEF Industry, Department of Biotechnology and Nanomedicine, NO-7465 Trondheim, Norway; 2 NTNU - Norwegian University of Science and Technology, Department of Biotechnology and Food Science, NO-7491 Trondheim, Norway

**Keywords:** Biotechnology, Industrial microbiology

## Abstract

Thraustochytrids of the genera *Schizochytrium* and *Aurantiochytrium* accumulate oils rich in the essential, marine n3 fatty acid docosahexaenoic acid (DHA). DHA production in *Aurantiochytrium* sp T66 was studied with the aim to provide more knowledge about factors that affect the DHA-productivities and the contributions of the two enzyme systems used for fatty acid synthesis in thraustochytrids, fatty acid synthetase (FAS) and PUFA-synthase. Fermentations with nitrogen starvation, which is well-known to initiate lipid accumulation in oleaginous organisms, were compared to fermentations with nitrogen in excess, obtained by oxygen limitation. The specific productivities of fatty acids originating from FAS were considerably higher under nitrogen starvation than with nitrogen in excess, while the specific productivities of DHA were the same at both conditions. Global transcriptome analysis showed significant up-regulation of *FAS* under N-deficient conditions, while the PUFA-synthase genes were only marginally upregulated. Neither of them was upregulated under O_2_-limitation where nitrogen was in excess, suggesting that N-starvation mainly affects the FAS and may be less important for the PUFA-synthase. The transcriptome analysis also revealed responses likely to be related to the generation of reducing power (NADPH) for fatty acid synthesis.

## Introduction

The marine, n3, long-chain polyunsaturated fatty acids (PUFA) docosahexaenoic acid (DHA) and eicosapentaenoic acid (EPA) are essential for humans, as well as for marine fish species. Fish oil is currently the main source of these fatty acids. As the production of fish oil cannot be further increased, microbial sources are expected to play an important role to supply the growing marine aquaculture, as well as the human population, with these essential n3 fatty acids. Thraustochytrids are unique because they can accumulate DHA in triacylglycerols stored in lipid droplets, and in particular the genera *Aurantiochytrium* and *Schizochytrium* are excellent producers of this fatty acid^1–3^. Lipid accumulation is initiated when an essential nutrient, e.g. nitrogen (N), limits cell division, similarly to the mechanisms for other oleaginous microorganisms.

Thraustochytrids use a standard fatty acid synthase (FAS) enzyme complex for synthesis of saturated fatty acids, mainly C14:0 and C16:0, while DHA and docosapentaenoic acid (DPA, C22:5 n6) is synthesized by a PKS-enzyme complex, the PUFA-synthase^4^. In addition to these four fatty acids, C16:1 and smaller amounts of unsaturated C18 and C20 fatty acids can be found in thraustochytrids, most likely synthesised from the FAS-products by desaturases and elongases. Despite many years of research, the mechanisms governing the relative activities of the two enzyme systems and the distribution of the carbon flow between them, are not known. This is essential knowledge needed to increase the DHA-productivity. Previous studies indicate that FAS is more easily affected by the cultivation conditions than the PUFA-synthase, such as temperature, oxygen levels and carbon limitation^1^. For instance, low oxygen levels increased the fraction of DHA in the lipids, but mainly due to reduced production of the fatty acids through FAS, while the DHA production was less affected^5–7^. However, in many of the studies of DHA-production by thraustochytrids dissolved oxygen has not been measured, which makes data interpretation difficult.

The two enzyme complexes used for fatty acid synthesis by thraustochytrids, FAS and the PUFA synthase, need the same precursors; acetyl-CoA and reducing power in the form of NADPH. Transcriptome analyses can provide information about which reaction steps in the biosynthesis of precursors, DHA and triacylglycerols (TAG) that are transcriptionally controlled. Genes involved in assembling the TAGs and synthesis of the lipid body membrane were strongly upregulated after N-depletion in the yeast *Yarrowia lipolytica*^8^, the fungi *Mortierella alpina*^9^, and two *Schizochytrium* species^10,11^, while *FAS* was not, or only slightly, upregulated. In the thraustochytrids and *Y. lipolytica*, no upregulation of genes involved in the generation of precursors (NADPH, acetyl-CoA) was observed. On the other hand, a strong upregulation of the NADPH-generating enzymes of the pentose phosphate cycle (PPP) was observed during lipid accumulation in *M. alpina*^9^.

The aim of the current study has been to enhance the understanding of basic mechanisms related to DHA synthesis in thraustochytrids. Fatty acid production rates and transcriptomes of *Aurantiochytrium* sp. T66^7,12^ were compared in fermentations where lipid accumulation was initiated by nitrogen-starvation and in fermentations with oxygen limitation and nitrogen in excess. Here, the term ‘starvation’ refers to conditions when a component is exhausted from the medium, and ‘limitation’ when a component is continuously supplied, but at growth-limiting rates giving no detectable concentrations in the medium. We used a completely defined medium with glutamic acid as nitrogen-source to get a distinct introduction of the N-starvation. Dissolved oxygen (DO) and pH were controlled, and online measurement of CO_2_ in the off-gas provided information about the metabolic activity and the exact time point for N-depletion. The annotation of genes^13^ putatively involved in precursor and lipid biosynthesis was manually curated, and the transcriptome data were used to elucidate responses related to fatty acid and precursor biosynthesis.

## Results

### Lipid accumulation and fatty acid profiles in batch fermentations with nitrogen starvation, and with nitrogen in excess and oxygen limitation

**Figure 1:**
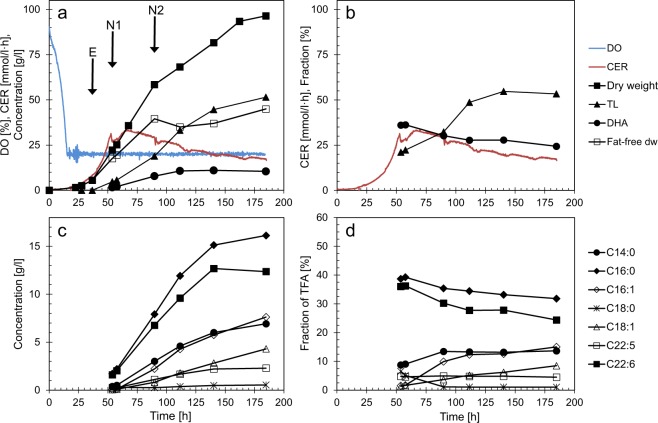
Cell growth and lipid production in fermentations with N-starvation. Data for one of four replicate fermentations are shown. Concentrations are corrected for dilution due to addition of glycerol and acid/base. E, N1, N2: Sampling points for transcriptome analysis. (**a,c**) Concentrations, dissolved oxygen (DO) and CO_2_ emission rate (CER). (**b,d**) Fractions (%): Total lipid (TL) of dry weight (dw); FA (fatty acids) of TFA (total fatty acids)

In experiments with nitrogen starvation, *Aurantiochytrium* sp. strain T66 was cultivated on a defined medium containing nitrogen as glutamate, sufficient to produce approximately 30 g/l catalytic biomass (‘fat-free’ cell mass), and with glycerol as carbon source in excess. Lipid accumulation started after approximately 50 h coinciding with the peak in CO_2_-emission (Fig. 1a,c), which reflects the glutamate depletion^7^. The final cell dry weight and lipid content were 95 g/l and 55%, respectively. The pH-control provided further information about metabolic shifts, as acid was added until ~50 h, followed by a period without need for pH-regulation until 66–70 h, and alkali addition during the rest of the fermentation. This is consistent with use of glutamic acid as carbon source and release of ammonia in the first phase of the fermentation. The fat-free dry weight continued to increase, from ~20 g/l at the onset of lipid accumulation, to ~35 g/l. No free ammonia was detected in the medium (detection limit ~2 mg/l). The increased fat-free dry weight after glutamate depletion therefore indicates that the cells utilised an intracellular pool of nitrogen in this period. As it appears from Fig. 1a,c, the volumetric fatty acid production rates were constant during the first 50–60 h of the lipid accumulation phase, until 100–115 h fermentation time. The production rates then decreased rapidly. The fractions of DHA and palmitic acid (C16:0) of total fatty acids (TFA) decreased slightly throughout the fermentation, in parallel with increasing fractions of C14:0, C16:1 and C18:1 (Fig. 1d). The synthesis of C16:1 and C18:1 was initiated after N-depletion and continued for a longer period than the synthesis of the saturated and polyunsaturated fatty acids. C18:1 occurred as the n7 isomer, not the more common n9 (oleic acid).

**Figure 2:**
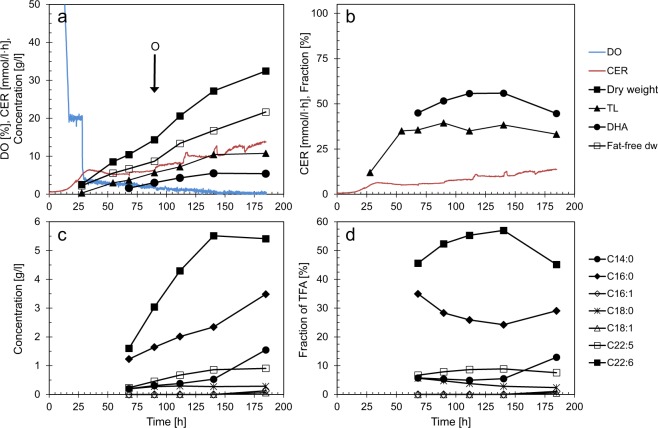
Cell growth and lipid production in fermentations with O_2_-limitation. Data for one of four replicate fermentations are shown. Concentrations are corrected for dilution due to addition of glycerol and acid/base. O: Sampling point for transcriptome analysis. (**a,c**) Concentrations, dissolved oxygen (DO) and CO_2_ emission rate (CER). (**b,d**) Fractions (%): Total lipid (TL) of dry weight (dw); FA (fatty acids) of TFA (total fatty acids)

For the fermentations with oxygen limitation, the conditions were identical to those with N-starvation for the first 28 hours. Then the automatic control of dissolved oxygen was turned off and the stirring lowered to a constant speed of 600 rpm. DO immediately dropped to 4% of saturation, followed by a further, slow decrease to the detection limit (Fig. 2a). The growth rate was lower than in the fermentations with N-starvation and no oxygen limitation, and the final cell density was only 33 g/l, and with ~37% total lipids (Fig. 2a,b). As opposed to N-starvation, fat-free dry weight increased during the whole fermentation. The DHA-fraction constituted 55% of TFA, compared to 28–30% during N-starvation, while the fractions of C16:0 and C14:0 were lower with O_2_-limitation than N-starvation (Fig. 2c,d). Glutamate was exhausted between the sampling points at 140 and 185 h. In this period, the concentrations and fractions of C14:0 and C16:0 increased, probably as a response to the N-depletion. No C16:1 or C18:1 was produced until the last sampling point, where traces (0.14 and 0.07 g/l, respectively) were detected. The volumetric fatty acid productivities were constant (Fig. 2a,c), while the specific productivity decreased due to the increasing fat-free dry weight (Table 1). The specific productivity of DHA was in the same range as for N-starvation, while the specific productivities of the FAS-generated fatty acids were far lower during oxygen limitation than during N-starvation.

**Table 1:** Specific productivities (q) of fatty acids under N-starvation and O_2_-limitation with N in excess.

Parameter	N-starvation	N in excess and O_2_-limitation
Fat-free dry weight [g/l]	20 → 35	7 → 17
qTFA [mg/(g·h)]	20 → 10	13 → 5
qFAS [mg/(g·h)]	12 → 6	4 → 1
qDHA [mg/(g·h)]	6 → 3	8 → 3

qTFA, qFAS, qDHA: Specific productivity of total fatty acids, fatty acids originating from FAS, and DHA, respectively.

The values are for the period 60–112 h for N-starvation and 80–140 h for O_2_-limitation and are calculated as volumetric productivities (mg/(l·h)) divided by the fat-free dry weight (g/l). The → indicate the changes during the calculation period.

### Global transcriptome analysis

Sampling for transcriptome analysis from the nitrogen starved fermentations was made during exponential growth (37 h, sample “E”), close to N-depletion (53 h, sample “N1”) and late in the rapid lipid accumulation phase (90 h, sample “N2”), as marked in Fig. 1. In the oxygen limited cultures, sampling for transcriptome analysis was made after 90 h (Sample “O”, Fig. 2). The total transcriptomes of the four fermentation samples E, N1, N2 and O, were analysed by RNA-sequencing (Supplementary Dataset). All abbreviations of genes and proteins are found in Table 2.

**Table 2:** Protein and gene abbreviations used in text and figures.

Abbreviation	Function
6PGD	6-phosphogluconate dehydrogenase
ACC	Acetyl-CoA carboxylase
ACL	ATP:Citrate lyase
ACO	Aconitase
ACOT	Acyl-CoA thioesterase
ACS	Acetyl-CoA synthetase
ACSL	Acyl-CoA fatty acid synthetase, long-chain
AGPAT	Acylglycerol-3-phosphate acyltransferase
AKGDH	α-ketoglutarate dehydrogenase
AMPD	AMP-deaminase
CS	Citrate synthase
d12D	Δ12 fatty acid desaturase
DGAT	Diacylglycerol acyltransferase
ELO	Fatty acyl-CoA elongase
FAS	Fatty acid synthase 1
FUM	Fumarase
G3PDH	Glycerol-3-phosphate dehydrogenase
G6PDH	Glucose-6-phosphate dehydrogenase
GABAT	γ-aminobutyrate aminotransferase
GAD	Glutamate decarboxylase
GDH	Glutamate dehydrogenase
GK	Glycerol kinase
GPAT	Glycerol-3 phosphate acyl transferase
GS	Glutamine synthetase
GUP	Glycerol uptake protein
ICL	Isocitrate lyase
IDH	Isocitrate dehydrogenase
MCAT	Malonyl-CoA acyltransferase
MDH	Malate dehydrogenase
ME	Malic enzyme
MS	Malate synthase
ORNT	Ornithine transporter
PAP	Phosphatidic acid phosphatase
PDH	Pyruvate dehydrogenase
PEPC	Phosphoenolpyruvate carboxylase
PFA	Polyunsaturated fatty acid synthase
PFAA	Polyunsaturated fatty acid synthase subunit A
PFAB	Polyunsaturated fatty acid synthase subunit B
PFAC	Polyunsaturated fatty acid synthase subunit C
PFAD	Phosphopantetheinyl transferase
PGL	6-phosphogluconolactonase
PGAM	Phosphoglycerate mutase
PYC	Pyruvate carboxylase
PYK	Pyruvate kinase
RPE	Ribulose-5-phosphate 3-epimerase
RPI	Ribose-5-phosphate isomerase
SCL	Succinate-CoA ligase
SDH	Succinate dehydrogenase
SSDH	Succinate semialdehyde dehydrogenase
TLDP	Lipid droplet protein

**Figure 3:**
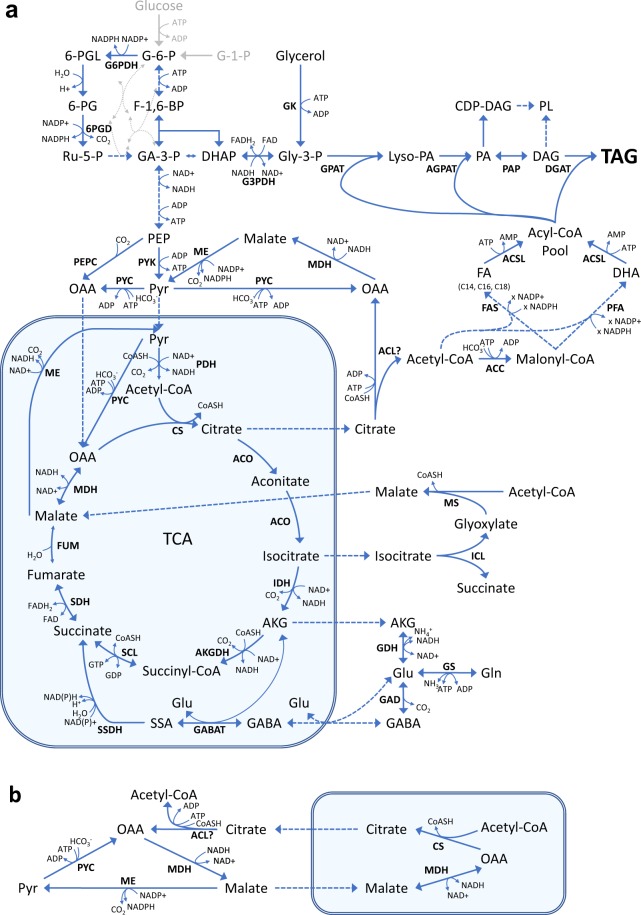
Metabolic pathways for synthesis of fatty acids and their precursors. The mitochondrion is boxed. (**a**) Overall picture; (**b**), close-up of the citrate/malate cycle and the transhydrogenase cycle. G-1-P: Glucose-1-phosphate; G-6-P: Glucose-6-phosphate; F-1,6-BP: Frucose-1,6-diphosphate; GA-3-P: Glyceraldehyde-3-phosphate; DHAP: Dihydroxyacetone phosphate; Gly-3-P: Glycerol-3-phosphate; 6-PGL: 6-phosphogluconolactone; 6-PG: 6-phosphogluconate; Ru-5-P: Ribulose-5-phosphate; PEP: Phosphoenolpyruvate; Pyr: Pyruvate; AKG: α-ketoglutarate; Gln: Glutamine; Glu: Glutamate; GABA: γ-butyrate; SSA: Succinate semialdehyde; OAA: Oxaloacetate; Lyso-PA: Lyso-phosphatidic acid; PA: Phosphatidic acid; CDP-DAG: Cytidine-diphosphate diacylglycerol; PL: Phospholipid; DAG: Diacylglycerol; TAG: Triacylglycerol; Abbreviations for enzymes and genes: See Table 2.

Genes related to the core metabolism and to lipid synthesis (Fig. 3; Supplementary Information, Table S1), as well as genes that were more than 8-fold differentially expressed between samples E, and N1, N2 and O, respectively, were manually annotated. The major fraction of the genes that were more than 8-fold differentially expressed, did not show extensive homology to any known proteins. This also applies to T6608762.2, which was the most highly expressed protein-encoding gene in N2 and O, with RPKM-values increasing from ~1000 in the exponential growth phase (E) to 60 000 in N2 and 55 000 in O. In the transition from exponential growth to N-depletion (sample N1), most of the downregulated genes seem to encode ribosomal proteins. Furthermore, two genes encoding key enzymes in amino acid biosynthesis were downregulated; phospho-2-dehydro-3-deoxyheptonate aldolase (T66008847.1 and T66004454.1) and aspartate semialdehyde dehydrogenase (T66005673.1 and T66008425.1). Most genes putatively encoding ammonium transporters were upregulated in N1, ranging from 1.7 to 23-fold. Moreover, the three most upregulated ones were more than five-fold downregulated under O_2_-limitation (O) where glutamate was in excess. These observations are consistent with utilisation of ammonium as nitrogen source in a period after depletion of glutamate. A few genes had very low transcript levels in all samples except N1. Most of these seem to encode flagella-related proteins. Also, a significant number of the genes encoding hypothetical proteins that predominantly were expressed in N1, contain motifs consistent with being involved in motility. This correlates with release of zoospores, which were observed just after N-depletion.

Since the aim of this study was to understand factors affecting the DHA biosynthesis, the main emphasis was the pathways needed for fatty acid and lipid biosynthesis (Fig. 3), and these were analysed in more detail.

### Expression of genes involved in generation of energy and reducing power

**Figure 4:**
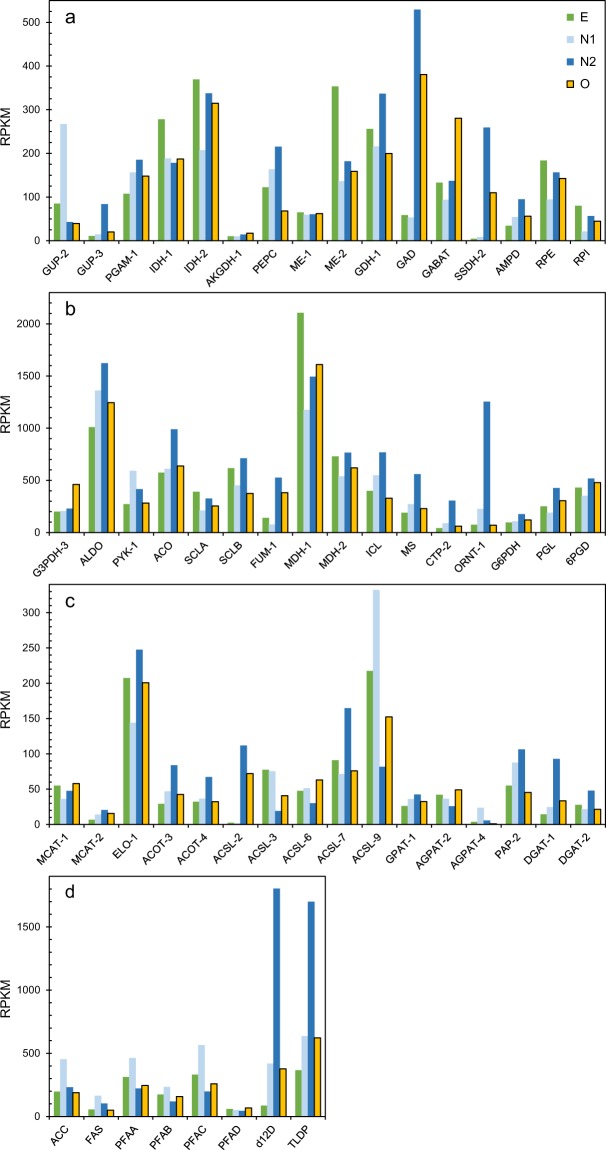
Expression of genes related to generation of energy and reducing power (**a,b**) and genes related to fatty acid and TAG-synthesis (**c,d**), as RPKM (Reads Per Kilobase Million). E: Exponential growth; N1: Glutamate exhaustion; N2: Rapid lipid accumulation phase; O: Oxygen limitation. E, N1 and N2 are from the same fermentations, while O is from parallel fermentations with similar conditions under exponential growth (E). Abbreviations: See Table 2.

Although glycerol was in excess during the fermentations, the preferred glycerol uptake facilitator protein apparently changed between the samples N1 and N2, as indicated by the shift in expression levels of the genes GUP-2 and GUP-3 (Fig. 4a). This is most likely related to the cessation of active cell growth between the N1 and N2 sampling points, see fermentations. None of the genes of the glycolysis or tricarboxylic acid cycle (TCA) had significant changes in expression levels between E and N2, while many of the TCA-genes were temporarily downregulated in the transition phase (N1) (Supplementary Information, Table S1). A gene annotated as a pyruvate kinase (T66000705) was an exception to this trend, it was upregulated in both N1 and N2.

Fatty acid synthesis requires reducing power as NADPH. The main source of NADPH for fatty acid synthesis in oleaginous microorganisms is assumed to be malic enzyme (ME)^14^, which converts malate to pyruvate as part of the transhydrogenase cycle (Fig. 3b). However, the genes encoding the enzymes of the transhydrogenase cycle were unchanged or downregulated in the N1 and N2 samples. On the other hand, genes encoding enzymes involved in generation of malate were upregulated in N2, such as *ICL* and *MS* of the glyoxylate cycle and the fumarase *FUM*-1 (Fig. 4b). *FUM*-1, which is predicted to be cytoplasmic by the prediction tool BaCelLo^15^, was 3.7-fold upregulated in the lipid accumulation phase. Two enzymes of the pentose phosphate pathway (PPP), glucose-6-P-dehydrogenase (G6PDH) and 6-phosphogluconate dehydrogenase (6PGDH), are other sources of NADPH. *G6PDH* was slightly upregulated in N2 (1.8-fold), while *6PGDH* was not affected. Interestingly, genes putatively encoding two steps of the γ-aminobutyric acid (GABA)-shunt, glutamate decarboxylase (GAD) and succinate semialdehyde dehydrogenase (SSDH), were strongly upregulated in the lipid accumulation phase, 9 and 62 times, respectively (Fig. 4a). The GABA-shunt bypasses two steps of the TCA-cycle, from α-ketoglutaric acid to succinate (Fig. 3), and SSDH may use NADP as cofactor. In summary, no identified genes encoding the main NADPH-forming enzymes were upregulated in the lipid accumulation phase, but significant upregulations were observed for some genes that indirectly may be involved in NADPH-generation.

### Expression of genes in pathways that generate malonyl-CoA

The initiating step in fatty acid synthesis is the conversion of acetyl-CoA to malonyl-CoA by the enzyme acetyl-CoA carboxylase (ACC). *ACC* was two-fold upregulated in N1 (Fig. 4d). In oleaginous microorganisms, the onset of lipid accumulation when nitrogen is exhausted, is triggered by an increased activity of AMP-deaminase (AMPD). Isocitrate dehydrogenase in the TCA-cycle is dependent on AMP. Reduced levels of AMP decreases the conversion of isocitrate, which in turn leads to accumulation of citrate^16^. The expression of *AMPD* increased 1.6-fold in sample N1 and further to 2.8-fold in the lipid accumulation phase (Fig. 4a). In all studied oleaginous microorganisms, citrate is transported out of the mitochondria and split to oxaloacetate and acetyl-CoA by the enzyme ATP:Citrate lyase (ACL). ACL is thus a key enzyme in oleaginous microorganisms, by providing both acetyl-CoA and malate (Fig. 3). However, no gene with any significant similarity to known ACL genes was identified in the T66 genome. The best hit was the probable succinate-CoA ligase (T66006817.1), which belongs to the same protein family as ACL.

### Expression of genes involved in fatty acid and triacylglycerol synthesis

**Figure 5:**
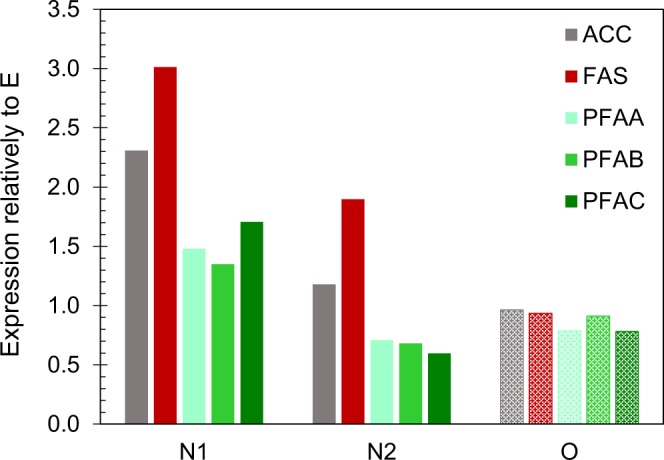
Relative expression levels of the genes *ACC*, *FAS* and PUFA-synthase. Abbreviations: See Fig. 4.

Genes encoding FAS and the three PUFA-synthase subunits A, B and C (PFAA, PFAB, PFAC) were all identified in strain T66. These genes had their highest expression levels at the transition stage (N1). For the PUFA-synthase genes, the expression levels were lower in the rapid lipid accumulation phase (N2) than under cell multiplication (E), while *FAS* maintained a higher expression level in the lipid accumulation phase than in the growth phase (Figs. 4d, 5). A gene that was annotated as a Δ12-desaturase had high expression levels and was the most upregulated (21-fold) in the lipid accumulation phase (Fig. 4d). A few other desaturases and elongases were also upregulated in the lipid accumulation phase (Supplementary Information, Table S1). Fatty acids and phospholipids are required for cell multiplication, while triacylglycerols are not. It is therefore likely that the amount of proteins that are specific for the synthesis of TAGs and lipid droplets will increase at the onset of TAG accumulation. Long-chain acyl-CoA synthases add CoA to the fatty acids as activation for TAG-synthesis or for fatty acid degradation. T66 contains many variants of these genes, probably encoding enzymes with different fatty acid-specificities. Several of these genes were upregulated, some at the transition and some in the lipid accumulation phase (Fig. 4c; Supplementary Table S1). An acyltransferase identified in *A. limacinum*^17^, which adds DHA to glycerol-3-phosphate, was also identified in T66 (GPAT-1, Fig. 4c). Diacylglycerol acyltransferase (DGAT) adds the third fatty acid to DAG to produce TAG. Two of the putative genes encoding this enzyme were considerably upregulated in the lipid accumulation phase (Fig. 4c). The gene encoding the thraustochytrid-specific lipid droplet protein (TLDP1)^3^, was five-fold upregulated in N2 (Fig. 4d). It is not unlikely that lipid degradation occurs simultaneously with lipid synthesis. Several genes encoding lipases, and enzymes related to β-oxidation of fatty acids, were considerably up-regulated in the lipid accumulation phase, and a few of them also in sample N1 (Supplementary Information, Table S1). Overall considered, the most interesting observation related to the expression of genes involved in fatty acid and triacylglycerol synthesis, was that *FAS* was upregulated during lipid accumulation when compared to the growth phase, while the PUFA-synthases were not.

### Comparison of gene expression between N-starvation and O_2_-limitation with N in excess

With only a few exceptions, the expression levels of genes involved in central metabolism and lipid synthesis were in the same range or lower under O_2_-limitation (sample O) than under N-starvation (sample N2) (Supplementary Information, Table S1). Among the few genes that had higher expression levels under O_2_-limitation, were some dehydrogenases in the glycolysis and TCA-cycle, as well as the γ-aminobutyrate aminotransferase (GABAT) of the GABA-shunt. Fatty acid and TAG-synthesising enzymes that had higher expression levels under O_2_-limitation than N-starvation, included the PUFA-synthase subunit C, some of the long-chain acyl CoA synthases (ACSL) and acylglycerol-3-phosphate acyltransferases (AGPAT) (Fig. 4; Supplementary Information, Table S1).

When considering the expression levels under oxygen limitation (O) compared to exponential growth (E), glutamate decarboxylase (GAD) and succinate semialdehyde dehydrogenase (SSDH) of the GABA-shunt were the most upregulated, 6.5 and 26-fold respectively, compared to 9 and 62-fold at N-starvation (Fig. 4a,b). The enzymes of the transhydrogenase-cycle (Fig. 3b) maintained the same expression levels independently of growth stage and conditions. Of the lipid-related genes, a putative trans-2-enoyl-CoA reductase (T66009285.1) was the most upregulated (7.7-fold) under O_2_-limitation, while the up-regulation under N-starvation was 5.1-fold. Most important, *FAS* and the PUFA-synthase genes maintained the same expression levels under oxygen limitation as under exponential growth. *ACC*, which generates malonyl-CoA from acetyl-CoA, followed the same pattern as *FAS* (Fig. 5).

## Discussion

Three main precursors are necessary for synthesis of triacylglycerols; malonyl CoA, reducing power in the form of NADPH, and glycerol 3-phosphate. The latter should be abundant in cells cultivated with glycerol as carbon source. The main sources of NADPH are PPP and malic enzyme, where ME has been assumed to play an essential role in oleaginous microorganisms^14^. In our study, the expression of the two identified ME-genes were unchanged, or lower, in the lipid accumulation phase than in the growth phase. Overexpression of ME did not increase the lipid production in the oleaginous fungi *Mucor circinelloides* and *M. alpina*, indicating that ME was not the rate-limiting step^18,19^. Other studies on *M. alpina* showed that PPP was essential for lipid accumulation^9^. In T66, the expression levels of G6PDH and PGL of PPP increased 1.8 and 1.7-fold, respectively, suggesting a contribution from PPP in generation of NADPH during lipid accumulation. However, a contribution from ME cannot be excluded despite no upregulation of the ME-genes. A role of ME is supported by the increased expression of genes involved in formation of malate, such as ICL and MS of the glyoxylate shunt from isocitrate to malate, and the cytosolic fumarase FUM-1. ICL and MS were also upregulated in the oleaginous yeasts *R. toruloides*^20^ and *Trichosporon oleaginosus*^21^ under N-limiting or N-depleted conditions, but not in *Y. lipolytica*, which lacks cytosolic ME^8^. Upregulation of fumarase after N-depletion has been shown for *Schizochytrium* sp.^11^, and also for the microalgae *Nannochloropsis*^22^ and the yeast *Rhodosporidium toruloides*^20^. Significantly increased volumetric fatty acid concentrations and content of the cell mass were obtained by feeding malate during the fermentation of *Schizochytrium* sp.^23^. This may indicate that the availability of malate is limiting. However, malate is needed not only as a substrate for ME, but also for an efficient transport of citrate out of the mitochondria via the citrate/malate cycle^24^ (Fig. 3b). The considerable up-regulation of the GABA-shunt may also be linked to NADPH-formation. We do not know whether the two dehydrogenases involved use NAD or NADP. Provided that the glutamate dehydrogenase uses NAD and the succinate semialdehyde dehydrogenase uses NADP, the shunt will serve as a transhydrogenation cycle. In *S. cerevisiae* it has been hypothesised that a role of the GABA-shunt is to reduce NADH-production in the mitochondrion and increase NADPH production in the cytosol^25^.

ATP:Citrate lyase (ACL) has been proposed to be a key enzyme in eukaryotic, oleaginous microorganisms^24^. We were not able to identify any gene with high similarity to known ACLs. Moreover, the published genomes for *A. limacinum, Aurantiochytrium sp. KH105, Hondae fermentalgiana* and *Schizochytrium sp*. CCTCC M209059 do not contain any genes homologous to known ACLs. However, *S. aggregatum* ATTC28209 encodes one such gene (JGI Schag1 Protein ID50064), and ACL activity has been reported for *Schizochytrium* sp.^26,27^. This could indicate that the ACLs of some thraustochytrids have low similarity to the characterized ones, or that some thraustochytrids lack this enzyme. The presence of an ACL in strain T66 is supported by the considerably increased expression of a citrate transporter in the lipid accumulation phase. However, cytosolic acetyl-CoA can in principle also be provided by mechanisms described for some non-oleaginous organisms, e.g. cytosolic acetyl-CoA synthase^28^ or carnitine-acetyl transporters^29^.

An observation that attracts attention, is a very strong up-regulation and a high expression level at N2 of the gene T66002957.1, which was annotated as a Δ12-desaturase. A similar observation was made by Ren *et al*.^11^. A Δ12-desaturase would convert C18:1 n9 to C18:2 n6. However, no C18:2, and only minor amounts of C18:1 n9, were detected in the lipids of *Aurantiochytrium* sp T66. In *Thraustochytrium* sp. ATCC 26185, an identical Δ12-desaturase gene was identified, but no products corresponding to the normal function of a Δ12-desaturase were formed^30^. The main monounsaturated fatty acids in T66 are C16:1 n7 and C18:1 n7. The concentration and fraction of these fatty acids, in particular C16:1, increased rapidly after N-depletion. C16:1 n7 is normally synthesised from C16:0 by a Δ9 desaturase, but we did not find any gene encoding proteins with significant homology to characterised Δ9 desaturases. Based on this, it seems likely that the protein encoded by T66002957.1 is involved in the biosynthesis of C16:1 n7, and possibly C18:1 n7.

Lipids accumulated also under oxygen-limitation with nitrogen in excess and the cell growth continuing throughout the fermentation. Lipid accumulation up to 30% of dry weight induced by O_2_ or phosphate limitation have previously been reported in thraustochytrids^12,31^. However, the mechanisms leading to lipid accumulation under other conditions than N-limitation is less characterised. The AMP-deaminase is a key enzyme in the initiation of lipid accumulation when N is exhausted but with no obvious role in lipid accumulation under O_2_-limitation. A slight up-regulation (1.7-fold) was observed, compared to 2.8-fold at N-starvation. Under N-starvation, the fatty acid synthesis rate decreased rapidly 50–60 hours after N-depletion. In the O_2_-limited fermentations with N in excess, the synthesis continued with constant rate for more than 100 hours. This may suggest enzyme degradation and a limited synthesis of new enzymes when N is exhausted from the medium.

None of the two enzyme systems for fatty acid synthesis were significantly up-regulated under O_2_-limitation, while *FAS* was considerably more upregulated than PUFA-synthase under N-starvation. The higher expression of *FAS* after N-depletion suggests that the synthetic capacity towards the FAS-products increased, while the PUFA synthase capacity was unchanged. This can explain the higher fraction and productivities of FAS-products under N-starvation than with N in excess, and that the specific DHA-productivities were similar at the two conditions. It is also consistent with a higher fraction of DHA under exponential growth than in the lipid accumulation phase. This has been reported for several thraustochytrids^32–34^. That the most critical factor for the DHA-fraction is N-starvation, not oxygen limitation, is in agreement with the studies of Chang *et al*.^5^. They showed that in O_2_-limited fermentations, the experiments with lowest oxygen supply and therefore delayed N-depletion, resulted in a lower lipid-concentration, but considerably higher DHA-fraction of the lipids than experiments with higher oxygen supply and N-starvation occurring close to the time-points for oxygen limitation.

## Conclusions

The lipids synthesised under N-starvation contained a higher fraction of the fatty acids originating from the fatty acid synthetase, and a lower fraction of DHA, than the lipids synthesised when nitrogen was in excess. This could be explained by an increased specific productivity of the FAS-products after N-exhaustion, while the specific productivity of DHA was unchanged. These results were supported by the transcriptome analyses, which showed that *FAS* was strongly upregulated after N-depletion, while the PUFA-synthase genes were only marginally upregulated. Neither of the two was upregulated in the fermentations with O_2_-limitation where nitrogen was in excess. Altogether, the results indicate that N-starvation mainly affects the production of the saturated and monounsaturated fatty acids and is less important for the DHA-production. Upregulation of genes coding for enzymes that generate malate, such as a cytosolic fumarase and the glyoxylate-shunt, suggests that malic enzyme is involved in the generation of NADPH needed for fatty acid synthesis. However, the downregulation of *ME* indicates that the catalytic capacity of ME is not a bottleneck. In summary, the present work has identified responses not previously reported or discussed for thraustochytrids. More investigations are needed in order to understand the importance of these findings for improving the DHA productivities.

## Materials and Methods

### Strain and cultivation conditions

*Aurantiochytrium* sp T66 (ATCC PRA-276^12,13^) was used in the study. The strain was preserved in 15% (v/v) glycerol at −80 °C. The fermentations were carried out in 3 l bioreactors with 1.5 l culture medium, which were inoculated with 60 ml (4%) actively growing culture. The fermentation medium contained initially (g/l): Glycerol 90, Sodium-glutamate hydrate 25, NaCl 14.4, CaCl_2_·2H_2_O 0.5, MgCl_2_ 0.8, KCl 0.4, KH_2_PO_4_ 4.0, Na_2_SO_4_ 3.0, maleic acid 5.8, Tris base 6.1, cyanocobalamin 5·10^–6^, thiamine·HCl 5·10^−5^, trace mineral solution 1.5 ml^12^, Sodium-ampicillin (0.3), and streptomycin sulphate (0.3). The antibiotics were used as a general precaution against bacterial contamination. Antifoam (Clerol FBA 622) was added as required. The bioreactors containing the medium were heat sterilized at 121 °C for 30 min. Phosphate was autoclaved separately, and the trace mineral and vitamin solutions were filter sterilized. The glycerol concentration was always kept above 15 g/l, by subsequent additions (3–4 times). The temperature was 28 °C, and pH was controlled at 7.0 ± 0.2 by addition of 3 N NaOH or 3 N H_2_SO_4_. The aeration rate was 0.3 vvm (0.45 l/min), and the dissolved oxygen was controlled at 20% of saturation by automatically adjustment of the stirring rate, if not stated otherwise. Inoculum for the fermentations was cultivated in 500 ml baffled Erlenmeyer flasks with 100 ml medium, on a rotary shaker (28 °C, 150 rpm). The medium was the same as the fermentation medium, but with glycerol reduced to 30 g/l. At each sampling point, approximately 60 ml culture broth was collected and distributed for analyses of dry weight, total lipids, fatty acid composition, and substrate consumption. Sampling for RNA-extraction was made at three time points. Product concentrations have been corrected for dilution due to addition of glycerol and H_2_SO_4_/NaOH. The correction factors for the end-point samples were 1.2–1.4, and measured concentrations in the bioreactors accordingly lower. The CO_2_-emission rate (CER) is calculated based on the fermentation start volume.

Four replicate fermentations were run at each of the two conditions (N-starvation and O_2_-limitation). Complete sets of analyses were carried out for three of these. For the transcriptomic data and other analytical data presented in text or tables, averages from the three fermentations are used.

### Analytical methods

#### On-line measurements

CO_2_ in the exhaust gas was measured by a mass spectrometer (Balzers Omnistar GSD 300 02), and the CO_2_ evolution rate (CER; mmol/l/h) was calculated. Temperature, pH, airflow, stirring rate and dissolved oxygen were recorded throughout the fermentations.

#### Cell growth, total lipids and substrate consumption

Two times 25 ml were centrifuged (3500 × g, 10 min) and washed once with an isotonic solution of NaCl; one sample was used for dry weight analysis, the other was frozen at −80 °C for determination of total lipids. Dry weight was determined by drying at 105 °C for 20–24 h. For determination of total lipids, the cell pellet was freeze dried, and lipids were extracted from heat treated and protease digested cell mass as described by Jakobsen *et al*.^7^. Supernatants after centrifugation were frozen (−20 °C) until analysis, and the concentrations of glycerol and glutamate were determined by HPLC, as previously described^7^, and ammonia was analysed by an enzymatic assay (Ammonia Assay Kit, Megazyme, Ireland).

#### Fatty acids

Ten ml culture was frozen directly (−80 °C). Fatty acids were quantified by LC/MS/MS (QQQ) after hydrolysis of the lipids to free fatty acids: KOH (5 M, 400 μL) was added to culture sample (100 µl, well homogenized) to a final concentration 4 M and incubated at 80 °C for 120 min. The free fatty acids were extracted into dichloromethane (2 mL) after acidification with 500 µl of 4 M H_2_SO_4_. The sample was vortexed for 60 seconds before centrifugation (4000 g, 10 min). 200 µl of the organic phase were transferred to a sample vial and the solvent evaporated under nitrogen at 60 °C. The samples were reconstituted in absolute ethanol and the vials were flushed with nitrogen before capping.

For LC/MS/MS analysis 1 µl of sample was injected on an Agilent 1290 LC system coupled to an Agilent 6490 QQQ mass spectrometer. The LC system was set up with an Ascentis Express column (15 cm × 2,1 mm, 2.7 µm, Supelco). Mobile phase A was a 25 mM aqueous solution of ammonium formate and mobile phase B was pure acetonitrile. The LC separation was performed with a gradient elution. The starting condition was 75% B, which was held for 0.5 min. Then a linear gradient to 100% B at 8.5 min and held for 1 minute. The mobile phase flow was 0.5 ml /min. The Agilent 6490 was equipped with an Agilent Jet Stream (AJS) ion source and operated in negative mode. Ion source parameters were: Nebulizer: 45 psi, gas temp: 250 °C, drying gas flow: 12 l/min, Sheat gas temp: 400 °C, sheat gas flow: 11 L/min, Nozzle voltage: 1500 V and capillary voltage: 3000 V. The mass spectrometer was operated in single ion monitoring mode (SIM). External standards were used for confirmation and quantification of the fatty acids.

### RNA isolation and sequencing

Five ml culture was centrifuged (3500 × g, 10 min at room temperature). All supernatant was carefully removed. The pellet was resuspended in deionized water to a standardised optical density and centrifuged (12 000 × g, 5 min), the supernatant removed, and the pellet frozen at −80 °C. RNA was isolated using the Spectrum™ Plant Total RNA Kit (Sigma). The On-Column DNase I Digest Set (SIGMA) was used to remove DNA. RNA integrity was evaluated on a Bioanalyzer 2100 with the Plant RNA Nano assay and software v. 1.3 resulting in RIN values of 8.9–9.5 for the 12 samples. The RNA concentrations were measured on a Qubit 2.0 Fluorometer using the Qubit^TM^ BR Assay Kit (Invitrogen), while RNA purity was evaluated by nanodrop spectroscopy, where a A260/280 ratio of ~2.0 generally was accepted as pure for RNA. The samples were shipped to BGI, Hong Kong, China for Illumina HiSeq-based RNA-seq in the 2·90 bp PE mode.

### Genome annotation

Functional annotation of the *Aurantiochytrium* sp. T66 draft genome was performed by BGI, Hong Kong, China combining de novo prediction and RNA-seq data. Adaptor sequences, reads with >5% unknown bases, or with >20% bases with below Q10 quality were removed by filtering, resulting in clean reads (Supplementary Information, Table S2), which were used for the functional annotation and for gene expression profiling.

Tandem repeats were identified using Tandem Repeats Finder (TRF)^35^ and transposable elements by a combination of a homology-based and de novo approaches^36^, using RepeatMasker^37^ and RepeatProteinMask, with the database of known repeats, Repbase^38,39^. RepeatModeler, Piler^40^, and LTR-Finder^41^ were used to build a de novo repeat library from the T66 genome, and to search for long terminal repeat retrotransposons (LTR). De novo gene prediction was performed based on the repeat-masked genome using AUGUSTUS^42^, SNAP^43^, and GlimmerHMM^44^. Homologous proteins of the species *Aureococcus anophagefferens*, *Nannochloropsis gaditana*, *Phaeodactylum tricornutum*, *Phytophthora infestans*, *Phytophthora sojae*, and *Thalassiosira pseudonana* were mapped to the genome using tblastn^45,46^ with an E-value cut-off 1·10^−5^. The aligned sequences as well as their corresponding query proteins were then filtered and passed to GeneWise^47^ to search for accurately spliced alignments. Source evidences generated from these approaches were then integrated by GLEAN^48^ to produce a consensus gene set. Cleaned RNA-seq reads were aligned against the genome using TopHat^49^ to identify candidate exon regions and the donor and acceptor sites. Then Cufflinks^50^ was used to assemble the alignments into transcripts. Finally, open reading frames were predicted based on the assembled candidate transcript sequences using HMM-based training parameters, and the GLEAN set was combined with the transcripts from the RNA-Seq to generate a final gene set.

Gene functions were assigned by blastP searches^46^ against the Swissprot and TrEMBL databases^51^, while Gene Ontology identifiers^52^ were assigned by InterProScan^53^. Finally the functional annotation of genes of interest was further curated by manual inspection and evaluation of blastn, blastp, and Position-Specific Iterated BLAST, PSI-blast, alignments with the hits from the NCBI nucleotide collection (nr/nt) and the non-redundant protein sequences (nr)^54^ in combination with the best hits from Swissprot and TrEMBL, in particular taking into consideration the fraction of the gene aligning to the hits (denoting all with less than 50% of the overall length as fragments) and the identity scores in the case of hits from multiple enzyme classes.

Transfer-RNAs (tRNAs) were found by tRNAscan-SE^55^. Small nuclear RNA (snRNA) and microRNA (miRNA) were identified by blastn followed by INFERNAL^56^ searches against the Rfam database^57^. Ribosomal RNA (rRNA), were found by blastN searches against rRNA.

During the manual curation of the genes of interest, some partial genes were identified, including T66005414.1 and T66005224.1 that both encoded partial polyunsaturated fatty acid synthase subunit A (pfaA) genes; T66000202.1, which encoded a partial polyunsaturated fatty acid synthase subunit C (pfaC), and T66002139.1, which encoded a partial glutamine synthetase. Additionally, no phosphopantetheinyl transferase (pfaD) gene had been called during the original annotation process. The reconstruction of these genes is described in Supplementary Information.

### RNA-seq transcriptome

The RNA-Seq analysis was performed in CLC Genomics workbench 11.0 (Qiagen). Clean reads were mapped against the T66_GeneModels by the RNA-Seq Analysis 2.16 tool in the “One reference sequence per transcript” mode with the expression value defined by Reads Per Kilobase Million (RPKM). Otherwise default parameters were used.

## Supplementary information

Supplementary Tables, Figures and Methods

Dataset 1

## Data Availability

The data discussed in this publication have been deposited in NCBI’s Gene Expression Omnibus^40^ and are accessible through GEO Series accession number GSE134374, (https://www.ncbi.nlm.nih.gov/geo/query/acc.cgi?acc=GSE134374).
