# Supplementary material for: Lipid and DHA-production in Aurantiochytrium sp. – Responses to nitrogen starvation and oxygen limitation revealed by analyses of production kinetics and global transcriptomes
Source: Sci Rep. 2019 Dec 19;9:19470. doi: 10.1038/s41598-019-55902-4 (PMC6923395; doi:10.1038/s41598-019-55902-4)
Supplement: Supplementary file 1 — Supplementary Tables, Figures and Methods [file 41598_2019_55902_MOESM1_ESM.pdf]

Tonje M. B. Heggeset, Helga Ertesvåg, Bin Liu, Trond E. Ellingsen, Olav Vadstein, Inga Marie Aasen

Lipid and DHA-production in *Aurantiochytrium* sp – Responses to nitrogen starvation and oxygen limitation revealed by analyses of production kinetics and global transcriptomes

Supplementary Tables, Figures and Methods

Table S1. Transcriptome data, expression as RPKM (reads per kilobase million), for the manually annotated genes

			RPKM				Fold difference			
**ID**	**Name**	**Abbr**	**E**	**N1**	**N2**	**O**	**N1/E**	**N2/E**	**O/E**	**O/N2**
**Glycerol uptake and conversion to glycerol phosphate**										
T66001838.1	Glycerol uptake facilitator protein	GUP-1	0.6	46.5	15.1	0.6	83.8	27.2	1.0	0.0
T66002465.1	Glycerol uptake facilitator protein	GUP-2	85	267	43	40	3.2	0.5	0.5	0.9
T66010800.1	Glycerol uptake facilitator protein	GUP-3	10	14	84	20	1.4	8.0	1.9	0.2
T66005684.1	Glycerol kinase	GK	594	678	429	612	1.1	0.7	1.0	1.4
T66002021.1	Glycerol-3-phosphate dehydrogenase	G3PDH-1	79	83	74	80	1.0	0.9	1.0	1.1
T66004387.1	Glycerol-3-phosphate dehydrogenase	G3PDH-2	6	14	6	5	2.4	1.0	0.9	0.9
T66006468.1	Glycerol-3-phosphate dehydrogenase	G3PDH-3	199	204	227	461	1.0	1.1	2.3	2.0
**Glycolysis and gluconeogenesis**										
T66001432.1	Phosphoglucomutase	PGM	374	372	293	305	1.0	0.8	0.8	1.0
T66010908.1	Phosphoglucomutase	PGM	153	192	217	193	1.3	1.4	1.3	0.9
T66007276.1	Hexokinase	HK	51	60	31	38	1.2	0.6	0.7	1.2
T66008242.1	6-phosphofructokinase	PFK	27	38	37	21	1.4	1.4	0.8	0.6
T66007108.2	6-phosphofructo-2-kinase/fructose-2,6-bisphosphatase	PFKFB	22	25	25	31	1.1	1.1	1.4	1.2
T66004362.1	Aldolase	ALDO	1009	1359	1622	1244	1.3	1.6	1.2	0.8
T66009200.1	Triosephosphate isomerase	TPI	495	445	547	538	0.9	1.1	1.1	1.0
T66010864.1	Triosephosphate isomerase	TPI	619	876	404	330	1.4	0.7	0.5	0.8
T66010775.1	Glyceraldehyde-3-phosphate dehydrogenase	GAPDH-1	81	47	67	45	0.6	0.8	0.6	0.7
T66011026.1	Glyceraldehyde-3-phosphate dehydrogenase	GAPDH-2	1978	1946	2217	2489	1.0	1.1	1.3	1.1
T66011142.1	Glyceraldehyde-3-phosphate dehydrogenase	GAPDH-3	908	1233	597	504	1.4	0.7	0.6	0.8
T66006855.2	Phosphoglycerate kinase	PGK-1	209	293	253	170	1.4	1.2	0.8	0.7
T66010974.1	Phosphoglycerate kinase	PGK-2	383	329	381	307	0.9	1.0	0.8	0.8
T66001704.1	Phosphoglycerate mutase	PGAM-1	107	156	185	148	1.5	1.7	1.4	0.8
T66009556.1	Phosphoglycerate mutase	PGAM-2	22	26	22	32	1.2	1.0	1.5	1.4
T66010905.1	Phosphoglycerate mutase	PGAM-3	580	531	657	484	0.9	1.1	0.8	0.7
T66006628.1	Enolase	ENO	1208	834	1102	860	0.7	0.9	0.7	0.8
T66000705.1	Pyruvate kinase	PYK-1	271	590	415	283	2.2	1.5	1.0	0.7
T66003792.1	Pyruvate kinase	PYK-2	45	46	69	50	1.0	1.5	1.1	0.7
T66006173.1	Pyruvate dehydrogenase E1 subunit alpha	PDHA	1012	596	513	495	0.6	0.5	0.5	1.0
T66011336.1	Pyruvate dehydrogenase E1 subunit beta	PDHB	902	697	575	547	0.8	0.6	0.6	1.0
T66002987.2	Pyruvate dehydrogenase E2 subunit	PDHC	458	245	249	273	0.5	0.5	0.6	1.1
T66002528.1	Pyruvate dehydrogenase x component	PDHX	460	303	290	310	0.7	0.6	0.7	1.1
T66008367.1	Dihydrolipoyl dehydrogenase	DLDH	905	568	698	970	0.6	0.8	1.1	1.4
**TCA**										
T66011598.1	Citrate synthase	CS	485	382	494	371	0.8	1.0	0.8	0.8
T66007190.4	Aconitase	ACO	572	609	988	638	1.1	1.7	1.1	0.6
T66002600.1	Isocitrate dehydrogenase	IDH-1	278	188	178	187	0.7	0.6	0.7	1.1
T66004470.1	Isocitrate dehydrogenase	IDH-2	369	207	337	315	0.6	0.9	0.9	0.9
T66006170.1	Alpha-ketoglutarate dehydrogenase	AKGDH-1	10	9	14	17	0.9	1.4	1.7	1.3
T66010195.3	Alpha-ketoglutarate dehydrogenase	AKGDH-2	312	197	278	210	0.6	0.9	0.7	0.8
T66010794.1	Alpha-ketoglutarate dehydrogenase	AKGDH-3	262	135	181	200	0.5	0.7	0.8	1.1
T66006817.1	Succinate - CoA ligase subunit alpha	SCLA	390	210	324	255	0.5	0.8	0.7	0.8
T66010760.1	Succinate - CoA ligase subunit beta	SCLB	616	450	710	375	0.7	1.2	0.6	0.5
T66002966.1	Succinate dehydrogenase	SDH-1	458	398	426	449	0.9	0.9	1.0	1.1
T66005536.1	Succinate dehydrogenase	SDH-2	912	761	865	704	0.8	0.9	0.8	0.8
T66009371.1	Succinate dehydrogenase	SDH-3	671	553	670	569	0.8	1.0	0.8	0.8
T66009538.1	Succinate dehydrogenase	SDH-4	444	345	364	401	0.8	0.8	0.9	1.1
T66000653.1	Fumarase	FUM-1	140	75	524	381	0.5	3.7	2.7	0.7
T66005350.1	Fumarase	FUM-2	165	117	86	99	0.7	0.5	0.6	1.2
T66000362.1	Malate dehydrogenase	MDH-1	2105	1174	1492	1609	0.6	0.7	0.8	1.1
T66007876.1	Malate dehydrogenase	MDH-2	729	538	765	620	0.7	1.0	0.9	0.8
**PPP**										
T66005578.1	Glucose-6-phosphate dehydrogenase	G6PDH	95	106	174	120	1.1	1.8	1.3	0.7
T66001999.1	6-phosphogluconolactonase	PGL	248	188	425	304	0.8	1.7	1.2	0.7
T66000832.1	6-phosphogluconate dehydrogenase	6PGD	429	350	516	478	0.8	1.2	1.1	0.9
T66008357.1	Ribulose-5-phosphate 3-epimerase	RPE	183	94	156	143	0.5	0.9	0.8	0.9
T66011653.1	Ribose-5-phosphate isomerase	RPI	80	21	56	45	0.3	0.7	0.6	0.8
T66011482.1	Transketolase	TKT	354	193	272	200	0.5	0.8	0.6	0.7
T66011516.1	Transaldolase	TAL	1388	940	1802	1912	0.7	1.3	1.4	1.1
**Associated reactions, cycles and shunts**										
T66007131.1	Phosphoenolpyruvate carboxylase	PEPC	122	163	215	68	1.3	1.8	0.6	0.3
T66000322.1	Fumarate reductase	FRD-1	21	18	34	31	0.9	1.6	1.5	0.9
T66006276.1	Fumarate reductase	FRD-2	308	490	347	238	1.6	1.1	0.8	0.7
T66007409.1	Fumarate reductase	FRD-3	39	53	23	32	1.4	0.6	0.8	1.4
T66001125.1	Pyruvate carboxylase	PYC	20	16	21	22	0.8	1.0	1.1	1.1
T66006070.1	Malic enzyme	ME-1	64	59	60	62	0.9	0.9	1.0	1.0
T66007895.1	Malic enzyme	ME-2	353	136	181	159	0.4	0.5	0.5	0.9
T66002129.1	Isocitrate lyase	ICL	398	547	767	329	1.4	1.9	0.8	0.4
T66003898.1	Malate synthase	MS	188	271	558	229	1.4	3.0	1.2	0.4
T66011704	Glutamine synthetase	GS	2	27	1	4	14.6	0.5	1.9	4.1
T66001169.1	Glutamate dehydrogenase	GDH-1	256	216	336	200	0.8	1.3	0.8	0.6
T66003915.1	Glutamate dehydrogenase	GDH-2	47	42	55	57	0.9	1.2	1.2	1.0
T66009186.1	Glutamate dehydrogenase	GDH-3	97	85	94	99	0.9	1.0	1.0	1.0
T66004665.1	Glutamate decarboxylase	GAD	58	53	529	381	0.9	9.1	6.5	0.7
T66003533.1	γ-aminobutyrate aminotransferase	GABAT	133	93	137	280	0.7	1.0	2.1	2.1
T66001194.1	Succinate semialdehyde dehydrogenase	SSDH-1	120	45	60	106	0.4	0.5	0.9	1.8
T66005295.1	Succinate semialdehyde dehydrogenase	SSDH-2	4.2	8.2	259	110	1.9	61.7	26.2	0.4
T66001332.1	Aspartate aminotransferase	AST	699	328	419	421	0.5	0.6	0.6	1.0
T66002557.1	Aspartate aminotransferase	AST	884	611	608	571	0.7	0.7	0.6	0.9
T66008840.1	AMP-deaminase	AMPD	34	54	95	56	1.6	2.8	1.7	0.6

			RPKM				Fold difference			
**ID**	**Name**	**Abbr**	**E**	**N1**	**N2**	**O**	**N1/E**	**N2/E**	**O/E**	**O/N2**
T66005762.1	Acetyl-CoA synthetase	ACS	45	35	29	17	0.8	0.6	0.4	0.6
T66008640.1	Succinyl-CoA D-citramalate CoA-transferase	SCCT	52	14	36	18	0.3	0.7	0.3	0.5
**Transport in/out of mitochondria**										
T66000025.1	Citrate transport protein	CTP-1	349	398	408	258	1.1	1.2	0.7	0.6
T66000466.1	Citrate transport protein	CTP-2	41	87	304	61	2.1	7.4	1.5	0.2
T66007495.1	Ornithine transporter, mitochondrial	ORNT-1	73	225	1252	70	3.1	17.2	1.0	0.1
T66007710.1	Ornithine transporter, mitochondrial	ORNT-2	14	41	114	10	3.0	8.4	0.8	0.1
T66011468.1	Citrate/ketoglutarate carrier protein	CCP	50	39	136	47	0.8	2.7	1.0	0.3
T66004483.1	α-ketoglutarate/malate carrier protein, mitochondrial	KGMT-1	20	25	4	8	1.2	0.2	0.4	2.0
T66004756.1	α-ketoglutarate/malate carrier protein, mitochondrial	KGMT-2	142	57	75	96	0.4	0.5	0.7	1.3
T66004735.1	Dicarboxylate/tricarboxylate transporter, mitochondrial	DCT	414	676	482	442	1.6	1.2	1.1	0.9
T66005256.3	Carnitine O-acetyltransferase	CAC-1	828	1000	288	505	1.2	0.3	0.6	1.8
T66006791.1	Carnitine O-acetyltransferase	CAC-2	72	79	218	66	1.1	3.0	0.9	0.3
T66009279.1	Mitochondrial carnitine/acylcarnitine carrier protein	CACT	284	204	281	168	0.7	1.0	0.6	0.6
**Fatty acid synthesis**										
T66005706.1	Acetyl-CoA carboxylase	ACC	196	452	231	189	2.3	1.2	1.0	0.8
T66010470.1	Malonyl-CoA acyltransferase	MCAT-1	55	36	47	58	0.7	0.9	1.1	1.2
T66001099.1	Malonyl-CoA acyltransferase, putative	MCAT-2	6	14	20	16	2.1	3.2	2.4	0.8
T66003188.1	Malonyl-CoA acyltransferase, putative	MCAT-3	15	14	8	16	0.9	0.6	1.1	1.9
T66001699.1	Acyl carrier protein	ACP	176	91	72	100	0.5	0.4	0.6	1.4
T66004311.1	Fatty acid synthase 1	FAS	54	163	103	50	3.0	1.9	0.9	0.5
T66011701	Polyunsaturated fatty acid synthase subunit A	PFAA	312	461	221	245	1.5	0.7	0.8	1.1
T66005413.1	Polyunsaturated fatty acid synthase subunit B	PFAB	173	233	118	158	1.3	0.7	0.9	1.3
T66011702	Polyunsaturated fatty acid synthase subunit C	PFAC	330	563	197	258	1.7	0.6	0.8	1.3
T66011703	Phosphopantetheinyl transferase	PFAD	58	50	43	68	0.9	0.8	1.2	1.6
T66002724.1	Δ6 fatty acid desaturase	d6D	4	12	12	2	2.9	2.8	0.5	0.2
T66002957.1	Δ12 fatty acid desaturase	d12D	85	417	1802	377	4.9	21.2	4.4	0.2
T66006792.1	Δ5 fatty acid desaturase	d5D	30	41	19	34	1.4	0.6	1.1	1.8
T66003476.1	Δ5 fatty acid desaturase	d5D	12	13	17	12	1.0	1.4	1.0	0.7
T66010139.1	Δ4 fatty acid desaturase	d4D	45	44	21	19	1.0	0.5	0.4	0.9
T66000527.1	Δ9 fatty acid desaturase	d9D	42	25	47	56	0.6	1.1	1.4	1.2
T66006246.1	Δ8 fatty-acid desaturase	d8D	77	72	48	54	0.9	0.6	0.7	1.1
T66003689.1	C18-Δ6/C18-Δ9/C20-Δ5 elongase	ELO-1	207	144	247	201	0.7	1.2	1.0	0.8
T66005597.1	Fatty acyl-CoA elongase	ELO-2	290	253	185	145	0.9	0.6	0.5	0.8
T66007915.1	Fatty acyl-CoA elongase	ELO-3	26	23	35	62	0.9	1.3	2.4	1.8
T66007922.1	Fatty acyl-CoA elongase	ELO-4	383	390	324	171	1.0	0.8	0.4	0.5
T66009340.1	C16-Δ9 elongase	ELO-5	27	28	6	7	1.0	0.2	0.2	1.2
T66006290.1	Trans-acting enoyl reductase	ER-1	432	99	146	284	0.2	0.3	0.7	2.0
T66006359.1	Trans-acting enoyl reductase	ER-2	67	49	38	77	0.7	0.6	1.2	2.0
T66007784.1	Trans-2-enoyl-CoA reductase	ER-3	6	8	7	11	1.3	1.0	1.7	1.7
T66009285.1	Trans-2-enoyl-CoA reductase	ER-4	18	55	94	142	3.0	5.1	7.7	1.5
T66011009.1	Enoyl-[acyl-carrier-protein] reductase	FabI	30	38	24	28	1.3	0.8	0.9	1.1
T66001907.1	Acyl-CoA thioesterase	ACOT-1	134	128	172	167	1.0	1.3	1.2	1.0
T66002260.1	Acyl-CoA thioesterase	ACOT-2	47	45	49	40	1.0	1.0	0.8	0.8
T66002759.1	Acyl-CoA thioesterase	ACOT-3	29	47	84	42	1.6	2.9	1.5	0.5
T66008091.1	Acyl-CoA thioesterase	ACOT-4	32	36	67	32	1.1	2.1	1.0	0.5
T66004579.1	Acyl-CoA fatty acid synthetase, short-chain	ACSS-1	6	9	27	14	1.4	4.2	2.1	0.5
T66004840.1	Acyl-CoA fatty acid synthetase, short-chain	ACSS-2	15	36	26	10	2.4	1.7	0.6	0.4
T66000024.1	Acyl-CoA fatty acid synthetase, long-chain	ACSL-1	37	24	16	29	0.7	0.4	0.8	1.8
T66001622.1	Acyl-CoA fatty acid synthetase, long-chain	ACSL-2	2	2	112	72	0.7	51.8	33.4	0.6
T66002738.1	Acyl-CoA fatty acid synthetase, long-chain	ACSL-3	77	75	19	41	1.0	0.2	0.5	2.2
T66002739.1	Acyl-CoA fatty acid synthetase, long-chain	ACSL-4	5	14	6	8	2.7	1.1	1.6	1.4
T66003686.1	Acyl-CoA fatty acid synthetase, long-chain	ACSL-5	38	53	16	11	1.4	0.4	0.3	0.7
T66005211.1	Acyl-CoA fatty acid synthetase, long-chain	ACSL-6	47	51	30	63	1.1	0.6	1.3	2.1
T66006986.1	Acyl-CoA fatty acid synthetase, long-chain	ACSL-7	91	71	164	76	0.8	1.8	0.8	0.5
T66007272.1	Acyl-CoA fatty acid synthetase, long-chain	ACSL-8	135	91	80	166	0.7	0.6	1.2	2.1
T66007597.1	Acyl-CoA fatty acid synthetase, long-chain	ACSL-9	217	332	82	152	1.5	0.4	0.7	1.9
T66009075.1	Acyl-CoA fatty acid synthetase, long-chain	ACSL-10	98	112	30	34	1.1	0.3	0.3	1.1
T66009076.1	Acyl-CoA fatty acid synthetase, long-chain	ACSL-11	87	131	121	62	1.5	1.4	0.7	0.5
T66009117.1	Acyl-CoA fatty acid synthetase, long-chain	ACSL-12	22	23	27	22	1.0	1.2	1.0	0.8
T66010236.1	Acyl-CoA fatty acid synthetase, long-chain	ACSL-13	2	25	8	1	14.2	4.9	0.5	0.1
T66010645.1	Acyl-CoA fatty acid synthetase, long-chain	ACSL-14	27	21	17	15	0.8	0.6	0.6	0.9
T66002859.1	Long-chain fatty acid transport protein (with ACSL)	FATP-1	90	122	99	28	1.4	1.1	0.3	0.3
T66004341.1	Long-chain fatty acid transport protein (with ACSL)	FATP-2	15	11	22	10	0.7	1.5	0.7	0.5
**TAG-synthesis**										
T66005184.1	Glycerol-3 phosphate acyl transferase	GPAT-1	26	36	42	32	1.4	1.6	1.2	0.8
T66005946.1	Glycerol-3 phosphate acyl transferase	GPAT-2	2	14	3	0	5.9	1.2	0.2	0.1
T66007924.1	Glycerol-3 phosphate acyl transferase	GPAT-3	43	68	55	48	1.6	1.3	1.1	0.9
T66008858.1	Glycerol-3 phosphate acyl transferase	GPAT-4	99	53	45	38	0.5	0.5	0.4	0.8
T66004032.1	Acylglycerol-3-phosphate acyltransferase	AGPAT-1	12	24	4	8	2.0	0.4	0.7	1.9
T66005810.1	Acylglycerol-3-phosphate acyltransferase	AGPAT-2	42	36	26	49	0.9	0.6	1.2	1.9
T66007568.1	Acylglycerol-3-phosphate acyltransferase	AGPAT-3	1	10	4	2	6.9	3.0	1.1	0.4
T66010013.1	Acylglycerol-3-phosphate acyltransferase	AGPAT-4	3	23	5	1	6.9	1.6	0.3	0.2
T66010664.1	Acylglycerol-3-phosphate acyltransferase	AGPAT-5	27	19	5	15	0.7	0.2	0.5	3.2
T66002159.2	Phosphatidic acid phosphatase	PAP-1	57	66	61	60	1.1	1.1	1.1	1.0
T66006416.1	Phosphatidic acid phosphatase	PAP-2	55	87	106	45	1.6	1.9	0.8	0.4
T66006417.1	Phosphatidic acid phosphatase	PAP-3	74	81	66	85	1.1	0.9	1.2	1.3
T66002778.1	Diacylglycerol acyltransferase	DGAT-1	14	24	93	34	1.7	6.6	2.4	0.4
T66003952.1	Diacylglycerol acyltransferase	DGAT-2	28	21	48	21	0.8	1.7	0.8	0.4
T66007335.1	Diacylglycerol acyltransferase	DGAT-3	111	86	86	87	0.8	0.8	0.8	1.0
T66011424.1	Diacylglycerol acyltransferase	DGAT-4	33	39	28	17	1.2	0.8	0.5	0.6
T66009334.1	Lipid droplet protein	TLDP	365	635	1698	622	1.7	4.7	1.7	0.4

ID	Name	Abbr	RPKM E	N1	N2	O	Fold difference N1/E	N2/E	O/E	O/N2
**PL-synthesis**										
T66010441.1	Ethanolamine kinase	EK	23	20	37	45	0.9	1.6	1.9	1.2
T66000212.1	Ethanolamine kinase	EK	145	129	164	92	0.9	1.1	0.6	0.6
T66000551.1	1-phosphatidylinositol-4-phosphate 5-kinase, family protein		14	17	17	29	1.2	1.2	2.0	1.7
T66003275.1	Phosphatidylinositol 3 and 4-kinase-like protein		6	34	14	2	6.1	2.4	0.4	0.2
T66001706.1	Diacylglycerol kinase	DGK	45	49	60	56	1.1	1.3	1.2	0.9
T66002626.1	Diacylglycerol kinase	DGK	2	13	5	1	8.5	3.6	0.5	0.1
T66007854.1	Diacylglycerol kinase	DGK	41	38	30	35	0.9	0.7	0.8	1.2
T66000277.1	Lysophosphatidylcholine acyl transferase	LPCAT	26	30	36	41	1.2	1.4	1.6	1.1
T66005465.1	Lysophosphatidylcholine acyl transferase	LPCAT	76	161	52	77	2.1	0.7	1.0	1.5
T66005769.1	Lysophosphatidylcholine acyl transferase	LPCAT	29	35	29	28	1.2	1.0	1.0	1.0
T66009152.1	Lysophosphatidylcholine acyl transferase	LPCAT	38	42	62	57	1.1	1.6	1.5	0.9
T66010977.1	Lysophosphatidylcholine acyl transferase	LPCAT	32	36	19	23	1.1	0.6	0.7	1.2
T66005776.1	Lysocardiolipin acyltransferase	LCLAT	73	114	204	130	1.6	2.8	1.8	0.6
T66003672.1	Phosphatidate cytidylyltransferase	CDS	79	112	92	89	1.4	1.2	1.1	1.0
T66008267.1	Phosphatidate cytidylyltransferase	CDS	42	44	33	49	1.1	0.8	1.2	1.5
T66010654.1	Choline/ethanolaminephosphotransferase	CEPT	109	78	60	65	0.7	0.6	0.6	1.1
T66002981.1	Choline/ethanolaminephosphotransferase	CEPT	90	49	36	68	0.5	0.4	0.8	1.9
T66004322.1	Phosphatidylethanolamine N-methyltransferase	PEMT	101	57	47	50	0.6	0.5	0.5	1.1
T66005702.1	Phosphatidylethanolamine N-methyltransferase	PEMT	34	39	45	34	1.1	1.3	1.0	0.8
T66005555.1	Ethanolamine-phosphate cytidylyltransferase	PECT	138	109	119	130	0.8	0.9	0.9	1.1
T66005046.1	Phosphatidylserine decarboxylase	PSD	61	56	33	40	0.9	0.5	0.6	1.2
T66009397.1	Phosphatidylserine decarboxylase	PSD	178	74	99	137	0.4	0.6	0.8	1.4
T66010026.1	Phosphatidylserine decarboxylase	PSD	42	44	43	54	1.0	1.0	1.3	1.3
T66008195.1	Phosphatidylserine decarboxylase	PSS	16	20	6	9	1.3	0.4	0.5	1.5
T66006631.1	Cardiolipin synthase	CRLS	14	13	8	25	1.0	0.6	1.8	3.1
T66009976.1	Cardiolipin synthase	CRLS	68	70	72	76	1.0	1.1	1.1	1.0
T66004308.1	Cyclopropane-fatty-acyl-phospholipid synthase		29	30	70	114	1.1	2.4	4.0	1.6
**β-oxidation**										
T66005991.15	Hydroxyacyl-CoA dehydrogenase trifunctional	HADHA	104	101	92	80	1.0	0.9	0.8	0.9
T66003371.1	Hydroxyacyl-CoA dehydrogenase trifunctional	HADHB	305	236	265	209	0.8	0.9	0.7	0.8
T66000450.1	Acyl-CoA dehydrogenase family protein	ACAD	458	382	448	361	0.8	1.0	0.8	0.8
T66004594.1	Acyl-CoA dehydrogenase family protein	ACAD	21	31	23	15	1.5	1.1	0.7	0.6
T66005144.9	Acyl-CoA dehydrogenase family protein	ACAD	62	49	15	45	0.8	0.2	0.7	2.9
T66007132.1	Acyl-CoA dehydrogenase family protein	ACAD	68	64	56	61	0.9	0.8	0.9	1.1
T66008866.1	Acyl-CoA dehydrogenase family protein	ACAD	33	30	32	27	0.9	1.0	0.8	0.9
T66009628.1	Acyl-CoA dehydrogenase family protein	ACAD	50	72	47	20	1.4	0.9	0.4	0.4
T66010787.1	Acyl-CoA dehydrogenase family protein	ACAD	117	92	85	192	0.8	0.7	1.7	2.3
T66010938.1	Acyl-CoA dehydrogenase family protein	ACAD	89	112	190	174	1.3	2.1	2.0	0.9
T66005078.1	Short-chain-enoyl-CoA hydratase	ECHS	5	40	6	3	8.1	1.2	0.6	0.5
T66006784.1	Short-chain-enoyl-CoA hydratase	ECHS	45	15	24	15	0.3	0.5	0.3	0.6
T66006999.1	Short-chain-enoyl-CoA hydratase	ECHS	123	72	101	100	0.6	0.8	0.8	1.0
T66003615.1	Enoyl-CoA hydratase domain-containing protein	ECH	27	13	11	26	0.5	0.4	0.9	2.4
T66003816.1	3-hydroxyacyl-CoA dehydrogenase	HCD	552	265	181	278	0.5	0.3	0.5	1.5
T66003861.1	3-hydroxyacyl-CoA dehydrogenase	HCD	166	108	101	166	0.6	0.6	1.0	1.6
T66001474.1	3-ketoacyl-CoA thiolase	ACAAT	267	238	161	184	0.9	0.6	0.7	1.1
T66001653.1	3-ketoacyl-CoA thiolase	ACAAT	373	244	212	208	0.7	0.6	0.6	1.0
T66004420.1	3-ketoacyl-CoA thiolase	ACAAT	100	147	60	97	1.5	0.6	1.0	1.6
T66007044.1	Enoyl-CoA delta isomerase	ECI	61	45	36	47	0.7	0.6	0.8	1.3
T66007045.1	Enoyl-CoA delta isomerase	ECI	239	149	140	202	0.6	0.6	0.8	1.4
T66008352.1	Enoyl-CoA delta isomerase	ECI	29	40	30	23	1.4	1.0	0.8	0.8
T66011012.1	Enoyl-CoA delta isomerase	ECI	31	27	15	18	0.9	0.5	0.6	1.2
T66004168.1	Acyl-coenzyme A oxidase	ACOX	8	16	24	21	2.0	3.0	2.6	0.9
T66006231.1	Acyl-coenzyme A oxidase	ACOX	114	244	408	66	2.1	3.6	0.6	0.2
T66003165.1	Bifunctional enzyme (Enoyl-CoA hydratase/ 3,2-trans-enoyl-CoA isomerase)	ECHP	133	67	75	59	0.5	0.6	0.4	0.8
T66005296.1	Bifunctional enzyme (Enoyl-CoA hydratase/ 3,2-trans-enoyl-CoA isomerase)	ECHP	51	54	43	54	1.1	0.8	1.1	1.3
**Lipases**										
T66001646.1	Triacylglycerol lipase	TGL	30	32	30	21	1.0	1.0	0.7	0.7
T66003504.1	Triacylglycerol lipase	TGL	20	24	56	28	1.2	2.8	1.4	0.5
T66005703.1	Triacylglycerol lipase	TGL	7	13	13	16	1.9	1.9	2.3	1.2
T66007930.1	Triacylglycerol lipase	TGL	10	10	9	14	1.0	0.9	1.5	1.6
T66008547.1	Triacylglycerol lipase	TGL	17	29	35	19	1.7	2.0	1.1	0.5
T66001057.1	Diacylglycerol lipase	DGL	52	38	29	37	0.7	0.6	0.7	1.3
T66004528.1	Diacylglycerol lipase	DGL	24	28	50	48	1.2	2.1	2.0	1.0
T66002561.1	Monoacylglycerol lipase	MGL	19	47	4	8	2.5	0.2	0.4	1.8
T66006391.1	Monoacylglycerol lipase	MGL	76	84	93	65	1.1	1.2	0.9	0.7
T66007376.1	Monoacylglycerol lipase	MGL	145	166	173	105	1.1	1.2	0.7	0.6
T66008893.1	Monoacylglycerol lipase	MGL	24	31	40	33	1.3	1.7	1.3	0.8
T66000209.1	Lipase	LIP	11	15	15	11	1.3	1.3	1.0	0.8
T66001369.1	Lipase	LIP	21	37	72	36	1.8	3.5	1.7	0.5
T66009381.1	Lipase	LIP	8	25	24	11	3.1	3.0	1.3	0.4
T66011638.1	Lipase	LIP	47	51	115	90	1.1	2.5	1.9	0.8
T66002193.1	Phospholipase	PL	27	12	32	29	0.4	1.2	1.1	0.9
T66002282.1	Phospholipase	PL	6	17	12	3	2.9	2.0	0.5	0.3
T66004034.1	Phospholipase D	PL	101	90	205	155	0.9	2.0	1.5	0.8
T66001944.2	Phospholipase D	PL	9	7	10	11	0.8	1.2	1.3	1.1

Table S2. RNA-seq data set used in the study

Sample	Code	Clean reads	Clean nucleotides
**E-1**	1-1	49,225,200	4,430,268,000
**E-2**	1-2	49,719,024	4,474,712,160
**E-3**	1-3	46,168,816	4,155,193,440
**N1-1**	2-1	48,955,990	4,406,039,100
**N1-2**	2-2	48,014,542	4,321,308,780
**N1-3**	2-3	48,844,540	4,396,008,600
**N2-1**	3-1	48,475,854	4,362,826,860
**N2-2**	3-2	49,465,088	4,451,857,920
**N2-3**	3-3	46,881,406	4,219,326,540
**O-1**	4-1	46,204,326	4,158,389,340
**O-2**	4-2	47,003,714	4,230,334,260
**O-3**	4-3	48,368,880	4,353,199,200

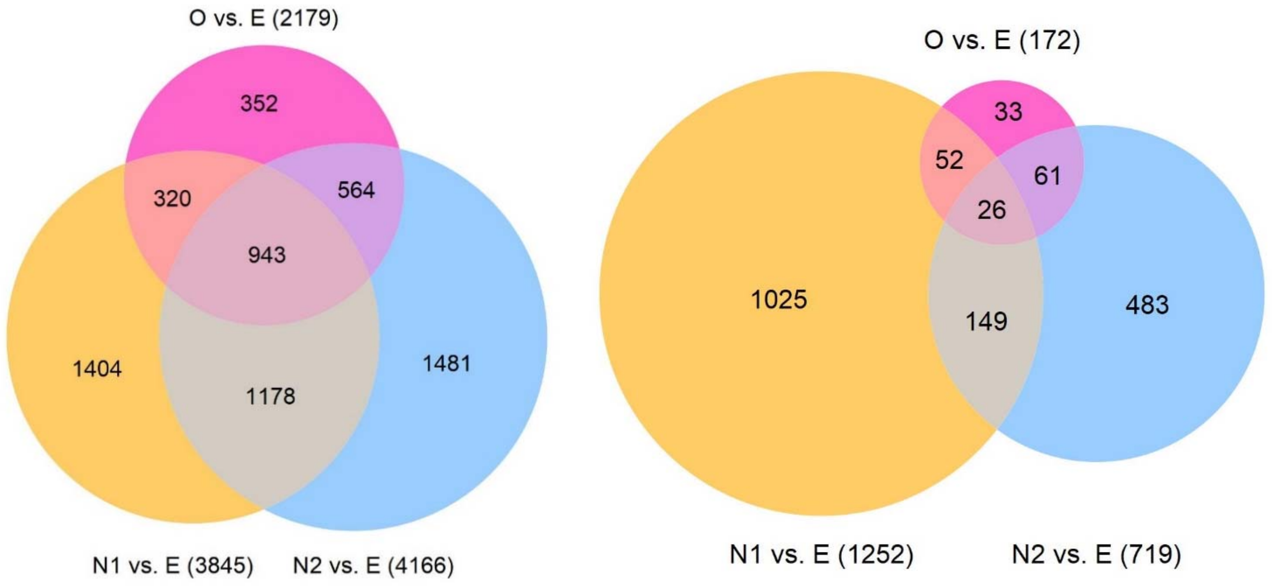

Figure S1. Venn diagram with the number of differentially expressed genes in samples N1, N2, and O compared to E (based CPM; counts per million reads mapped). Cut-offs of 2-fold (left) and 8-fold (right) regulation. E: Exponential growth (37 h); N1: Transition (54 h); N2: Lipid accumulation (90 h)

The differential expression was calculated using the "Differential Expression for RNA-Seq 1.0" tool comparing between all group pairs, combining the 3 replicates in groups and with no additional controls set. Venn diagrams were generated from the differential expression files by the "Create Venn Diagram for RNA-Seq 0.1" tool.

Methods

Reconstruction of incomplete genes

During the manual curation of the genes of interest some partial genes were identified, including T66005414.1 and T66005224.1 that both encoded partial polyunsaturated fatty acid synthase subunit A (pfaA) genes; T66000202.1, which encoded a partial polyunsaturated fatty acid synthase subunit C (pfaC), and T66002139.1, which encoded a partial glutamine synthetase. Additionally, no phosphopantetheinyl transferase (pfaD) gene had been called during the original annotation process. An annotated draft genome assembly of *Hondaea fermentalgiana* FCC1311 (Genbank: BEYU00000000.1) was recently published and in addition a draft genome assembly with 9 annotated genes related to fatty acid synthesis is available of *Thraustochytrium* sp. ATCC 26185 (Genbank: MUFY01000000; KX651612- KX651618; AB762292- AB762293) (Meesapyodsuk and Qiu, 2016[1]; Zhao et al 2016[2]). The pfaA encoded by KX651612 (showing 100% identity to bp. 200371-203795 of Genbank: KQ758867 and 99.90% identity to bp. 113505-110610 of Genbank:KQ758864), the pfaC encoded by KX651614 (showing 99.97% identity to bp 526485-522565 of Genbank: KQ758850), and the pfaD encoded by KX651615 (showing 99.74% identity to bp 16311163-1631929 of KQ758850) were used as BLAST queries to reconstruct the corresponding genes from *Aurantiochytrium* sp. T66. Due to the very high sequence similarity between the two strains, missing sequences were adapted from the *Thraustochytrium* sp. ATCC 26185 sequences and corrected with RNA-Seq read mapping. Similarly the glutamine synthetase was reconstructed by fusing bp 1518-2104 from Genbank: LNGJ01005523 to bp 8203375 8203835 of KQ758850 based on alignment to the *H. fermentalgiana* FCC1311 (Genbank: BEYU01000128 gene FCC1311_089412; protein id: GBG32716) sequence (89.06% identical) and to bp 1-1047 of the *Thraustochytrium* sp. ATCC 26185 contig MUFY01000151 (99.9% identical). The resulting genes - T66011701 encoding pfaA, T66011702 encoding pfaC, T66011703 encoding pfaD, and T66011704 encoding glutamine synthase replaced the partial genes and were included in the template file for RNA-Seq analysis.

---

[1] Meesapyodsuk, D. & Qiu, X (2016) Biosynthetic mechanism of very long chain polyunsaturated fatty acids in *Thraustochytrium* sp. 26185. *J. Lipid Res.* **57**, 1854-1864.

[2] Zhao, X. M., Dauenpen, M., Qu, C. M. & Qiu, X. (2016) Genomic analysis of genes involved in the biosynthesis of very long chain polyunsaturated fatty acids in Thraustochytrium sp 26185. Lipids 51, 1065-1075.
